# The effect of cassava and wheat starches complexation with selected fatty acids on their functional properties

**DOI:** 10.1007/s13197-021-05153-x

**Published:** 2021-06-04

**Authors:** Karolina Królikowska, Sławomir Pietrzyk, Henryk Pustkowiak, Kinga Wolak

**Affiliations:** 1 Department of Food Analysis and Evaluation of Food Quality, Faculty of Food Technology, University of Agriculture, Balicka 122 Str, 30-149 Krakow, Poland; 2 Department of Cattle Breeding, Faculty of Animal Science, University of Agriculture, 29 Listopada 46 Str, 31-425 Krakow, Poland

**Keywords:** Starch lipid complexes, Pasting properties, In vitro digestibility, Water solubility

## Abstract

The aim of the study was to evaluate the impact of selected fatty acids: palmitic, stearic or oleic on functional properties of cassava and wheat starches. Effectiveness of complexation procedure was evaluated by determination of complexing index, lipids content and fatty acid composition. Native starches and their complexes with fatty acids were analyzed for water binding capacity and solubility in water, in vitro digestibility, pasting characteristic and rheological properties. On the basis of the obtained results it was found that values of complexing index values for both starches complexed with oleic acid were remarkably higher than those determined in palmitic or stearic counterparts. Starches complexation with fatty acids resulted in a significant increase in the percentage share of used fatty acid in total fatty acid composition. Presence of oleic acid in cassava starch significantly limited its water binding capacity and solubility in water. Complexation with all fatty acids used in the study resulted in an increase in final viscosity and rheological stability of cassava starch pastes. Wheat starch complexes with fatty acids were characterised by higher pasting temperatures as compared to non-complexed counterpart, with the greatest effect observed for starch–oleic acid complex. In most cases complexation of starches with fatty acids contribute to an increase in contents of resistant starch fraction. Analysis of rheological model parameters revealed that procedure of oleic acid complexation increased by twice the values of consistency coefficient, and significantly decreased flow index of both wheat and cassava starches.

**Supplementary Information:**

The online version contains supplementary material available at 10.1007/s13197-021-05153-x.

## Introduction

Fatty acids as a part of triacyloglycerols are the principal contributors of dietary fat in human diet. A major methabolic role of fatty acids is as a source of energy, as a substrates for cell membrane biogenesis or as a precursors of many intracellular signaling molecules (Lunn and Theobald 2006). In food processing lipids and fatty acids are commonly used in the formulations of food products in order to improve their quality and sensory attractiveness. Therefore, the growth of scientific interest in the field of analysis of composition, and role of lipids in foods is observed. Liu et al. (2002) performed modification of the proportions of fatty acids (especially increase in oleic and stearic acids contents) in oilseeds in order to improve their nutritional value, and functional properties. It was observed that increase in the level of stearic or oleic acids in soybean oil improving its stability during storage (preventing oxidative stress) and utility for baking applications (Pantalone et al. 2002). Moreover, in food processing palmitic acid may be used as a constituent of edible films and coatings which determines barrier properties of the material with regard to water vapour, oxygen, carbon dioxide and lipid transfer in food systems (Guilbert et al. 1996). However, lipids including fatty acids are food ingredients sensitive to environmental stress. Food processing or storage contributed to loss in amounts of this substances and their bioactivity. Those undesirable changes negatively influence the shelf-stability and sensory parameters of the final product (Bakry et al. 2016). Therefore, tools to preserve the beneficial properties of these active substances are still in a scope of scientists interest. Furthermore, in order to improve nutritional value of food products the supplementation of desire lipids became a commonly used approach in the food production. One of the techniques which can be used for fatty acid supplementation in food is their immobilization in starch in form of the starch–lipid complexes.

Amylose fraction of starch is known as a linear polymer, but in space it frequently forms a helical structure. The helices structure is stabilized mostly by hydrogen bonds and van der Waals contacts. Generally, hydroxyl groups of glucosyl residues are located on the outer surface of the helix, while the internal cavity is a hydrophobic tube. Therefore, amylose has an ability to form a molecular inclusion complexes (or guest–host complexes) with a hydrophobic molecules including fatty acids (Panyoo and Emmambux 2016). Complexation of starch with other molecules is concerned as an alternative to conventional encapsulation techniques. Some studies demonstrated that, the quest molecule in amylose inclusion complexes were effectively protected by either oxidation or thermal degradation (Kawai et al. 2012; Marinopoulou et al. 2016; Li et al. 2019). So far, in order to enhance polymer ability to complexing with lipids native starch was subjected to gelatinization or debranching process. However, such procedures contributed to loss of the thickening properties of the starch and significantly limited its application in food production. Therefore, some studies on native starch complexation with fatty acids at a temperature below pasting temperature were performed. Chang et al. (2013) shown that swelled normal maize starch was easy to form complexes with lauric acid. Similar observations were made by Cui and Oates (1999) who indicated formation of amylose lipid complexes after native sago starch incubation with selected monoglicerides.

The presence of fatty acids in starch affected properties of obtained starch derivatives. However, the direction of changes depends on factors like: botanical origin of starch, contents of amylose and amylopectin components, saturation and length of the chain of fatty acid used for the complexation. Fewer studies concern the influence of the length and saturation of the fatty acid on the properties of native starch–fatty acid samples. Wang et al. (2016) demonstrated that formation of amylose-saturated fatty acid complexes in normal starch is affected by the length of fatty acid used for complexation. They indicated that short chain fatty acids can form complex more easily than long chain fatty acids. Additionally, these authors showed that effectiveness of complex formation with waxy starch was much lower than that of normal starch. In turn, studies of Arik Kibar et al. (2014) proved that addition of six different fatty acids caused a diverse effect on functional properties of corn starch.

The origin of starch influences not only its chemical composition, thermal, functional or physical properties, but also its susceptibility to form complexes with lipids. Zhou et al. (2007) demonstrated that complexation of stearic or linoleic acids with granule rice starch influenced gelatinization and pastes retrogradation processes. But, there are very limited reports on the complex formation between wheat or cassava starches and fatty acids, and the properties of obtained starch derivatives. Both wheat and cassava are extensively cultivated crops in the world, which are widely consumed as a staple food or additive and are an important starch resources. Cassava starch (CS) due to its unique thickening properties, high purity and ability to form clear viscous pastes is widely used in food industry (Zhu 2015). While, wheat starch (WS) forms a viscoelastic pastes when heated in water, and plays an important role in texture and quality of food products, especially dough and bread (Yu et al. 2018). Starch interactions with fatty acid affect both, physicochemical and functional properties of the polymer. Obtained starch–lipids complexes exhibited modified susceptibility to enzymatic hydrolysis, rheological and textural properties or storage stability. Therefore, the aim of the study was to evaluate the impact of selected fatty acids on functional properties of cassava and wheat starches. Additionally, investigation of the susceptibility of the native starches for fatty acid complexation and the functional properties of the obtained derivatives may extend the spectrum of their applications in the food industry as a fatty acid carriers.

## Materials

Cassava(C*CreamGel 70001) and wheat (C*Gel 20006) starches provided by Cargill BV (Sas Van Gent, Netherlands) were used in this study. Amylose contents in cassava and wheat starches were 26.60 ± 1.89 and 35.05 ± 0.78, respectively. The contents of protein and ash were 0.37% ± 0.01 and 0.16% ± 0.01 in cassava starch, or 0.35% ± 0.02 and0.19% ± 0.01 in wheat starch. Oleic (OA), stearic (SA), and palmitic (PA) acids were purchased from Chempur (Piekary Śląskie, Poland). α-amylase from porcine pancreas (type VI-B) and amyloglucosidase from *Rhizopusmold* (A-7255) were obtained from Sigma-Aldrich Chemical Company (St. Louis, MO, USA). All other chemicals used in the study were of analytical grade.

### Preparation of starch–fatty acid complexes

The complexation process of starch with fatty acids (palmitic, stearic, oleic) was carried out following a procedure described by Wang et al. (2016). Fatty acid in amount of 1.5 mmol was dissolved under magnetic stirring in 30 mL of absolute ethanol. Then, 10 g of dry weight (d.w.) of starch (cassava or wheat) was mixed with fatty acid–ethanol solution. The suspension was continuously stirred in a fume hood till the complete evaporation of ethanol (approximately 48 h).Finally, the resulting powder of starch–fatty acid complex was stored.

## Methods

### Determination of effectiveness of the complexation process

Starch–fatty acid complexes were examined for the complexing index (CI) according to Wang et al. (2016). Initially, 0.4 g of starch–fatty acid sample was dispersed in 10 ml of distilled water and then heated for 25 min in a water bath at 95 ºC ± 0.5º with occasional shaking. Afterwards, the gelatinized starch paste was cooled to room temperature, mixed with 20 ml of distilled water and the whole mixture was vortexed for 2 min. An aliquot of 500 µl of the starch solution was withdrawn, diluted in 15 ml of distilled water, followed by the addition of 2 ml of iodine solution (2.0% KI and 1.3% of I_2_ in distilled water). The maximum absorbance of analyzed starch derivatives with iodine complexes was measured at 690 nm. Native cassava and wheat starches were used as reference sample. CI values were calculated as follows (1):

1 $$ CI = \frac{{(A_{control} - A_{sample} )}}{{A_{control} }} \cdot 100 \left( \% \right) $$

where: *A*_*control*_ is the absorbance of the solution of starch without added fatty acids, and *A*_*sample*_ is the absorbance of the solution of starch complexed with fatty acids.

### Total lipids content and fatty acids composition

In order to determination of total lipids content the lipids were Soxhlet extracted from tested starch preparations in a Büchi B-811 extractor (Warsaw, Poland) with petroleum ether at 80 °C. The amount of total lipids was expressed as a sum of the extracted lipids after evaporation of organic solvent from the recipients and its drying to a constant weight.

Prior to analysis of fatty acid composition, lipids were extracted from tested samples using chloroform–methanol mixture (2:1 v/v) according to slightly modified Folch et al. (1957) procedure. Starch–fatty acid sample (1 g; dry weight basis) was weight and mixed with 15 mL of chloroform–methanol mixture. The whole suspension was homogenized for 10 min at 5000 rpm using homogeniser MPW-120 (MPW Med. Instruments, Warsaw, Poland), and after 5 min pause homogenization was repeated for 5 min at 1000 rpm. Afterwards, the obtained mixture was filtered through filter paper to the regular cylinder and filled with extraction mixture up to 15 mL, followed by the addition of 3 mL of 0.74% (w/v) KCl solution. After phases separation the alcohol–water phase was removed. The remaining chloroform phase was washed 3 times using 2 mL of the chloroform–methanol-0.74% KCl (3:48:47, v/v/v) mixture. Finally, the chloroform phase was recovered, dehydrated with anhydrous sodium sulphate (Na_2_SO_4_) and dried using nitrogen at 45 °C.

Afterwards, fatty acids present in extracted lipids were esterified according to standard analytical method 991.39 (AOAC 1995). The composition of fatty acids was determined with a gas chromatograph (GS) Trace Ultra (Thermo Electron, Milano, Italy) with a flame ionization detector (FID) equipped with column Supelcowax 10 (L × I.D. 30 m × 0.25 mm, d_f_ 0.25 μm, Sigma Aldrich Chemical Company, St. Louis, MO, USA). The temperature of the column was programmed from 160 °C to 210 °C at 3 °C/min, then kept constant at 210 °C for 25 min. A helium was used as a carrier gas and a flow rate was set to 1 mL/min.The injector and detector temperatures were 220 °C and 250 °C, respectively.

### Determination of water binding capacity (WBC) and solubility in water (SW)

Water binding capacity and solubility in water of tested starch–fatty acid complexes at temperatures of 60 °C, 70 °C and 80 °C were determined by the method described by Li et al. (2016). Starch water suspensions (1.5%, d.w.) prepared in screw-cap test tube were heated at water bath at temperature of determination for 1 h with intermittent shaking. The tubes were then cooled to room temperature. After centrifugation at 5600× *g* for 30 min, the supernatant was poured out in a glass pan and precipitate was weighed (W_p_). The supernatant was dried in an air oven at 100 °C to constant weight (W_s_). The water binding capacity of starch (2) and its solubility in water (3) were calculated as follows:

2 $$ {\text{WBC}}\frac{{W_{p} }}{{Weightofstarch \cdot \left( {100 - SW} \right)}}\left( {g/gd.w.ofstarch} \right) $$

3 $$ {\text{SW = }}\frac{{W_{s} }}{Weightofstarch } \cdot 100 \left( \% \right) $$

### Pasting characteristic

Effect of cassava or whet starches complexation with fatty acid on their pasting characteristic were determined by a Rapid Visco Analyzer (RVA) (Perten Instruments, Hägersten, Sweden). The pasting parameters of 8% (w/w) starch suspensions were measured using the following temperature program. The slurries were first held at 50 °C for 1 min, then heated to 95 °C within 5.5 min, and held at 95 °C for 5 min. The hot samples were subsequently cooled to 50 °C within 5 min, and maintained at 50 °C for another 5 min. The agitation speed of paddle during the run was 160 rpm. Pasting parameters including: peak viscosity at heating (PV), hot paste viscosity at 95 °C (HPV), final viscosity at 50 °C (FV), breakdown value (BD = PV–HPV), setback value (SB = FV–HPV), and pasting temperature (P_t_) were obtained from viscograms.

### In vitro digestibility

The in vitro starch digestion of native starches and their complexes with fatty acids was determined following Zhang et al. (2017) method with minor modification. Starch samples (100 mg) were dispersed in 2.5 ml of water, pasted in shaking boiling water bath for 20 min, then cooled. Afterwards, starch pastes were incubated in 37 °C with 5 ml of phosphate buffer (pH 5.2). After equilibration of samples temperature, 2.5 ml of enzyme mixture (α-amylase and amyloglucosidase mixed in proportion of 120U/80U/mL) was added and the starch digestion started. At regular intervals, of 20 or 120 min of incubation, 250 µl of samples were withdrawn, and mixed with 4 ml of 70% (v/v) ethanol solution. The reducing sugar content was measured using 3,5-dinitrosalicylic acid (DNS). Contents of three different starch fractions were calculated: rapidly digestible starch (RDS), which corresponds to the amount of starch hydrolysed after 20 min; slowly digestible starch (SDS), corresponding to the amount of starch hydrolysed between 20 and 120 min; and finally, resistant starch (RS), which is the totals starch of the food minus the amount of starch hydrolysed within 120 min.

### Rheological measurements

The rheological properties of 5% (w/w) starch pastes were evaluated by shear flow curves measurement using a rotational rheometer Rheolab QC (Anton Parar, Germany) with system of coaxial cylinders (cup diameter: 28.92 mm, bob diameter: 26.66 mm). Tests were controlled by Rheo Compass software (Anton Parar, Germany). Starch pastes were obtained by heating the 5% (m/m) starch suspension in boiling water bath for 30 min at constant stirring. Prepared pastes were placed in measuring system, relaxed and thermostated at 50 °C. Afterwards, samples were measured at a shear rates ranging between 1 s^−1^ and 300 s^−1^. Obtained data were fitted to Power-Law Model (4):

4 $$ \tau = K \cdot \dot{\gamma }^{n} $$

where τ – shear stress, Pa; K – consistency index, Pa s^n^;$\dot{\gamma }$ – shear rate, s^−1^; and n – flow behaviour index.

### Statistical analysis

All determinations were performed at least in triplicate. The obtained data were evaluate by one-factor analysis of variance, and significant differences were determined by Fisher’s test at significance level of 0.05. Pearson correlation coefficients (*r*) were calculated to describe the association between selected starch properties.

## Results and discussion

### Effectiveness of the complexation process

Presence of lipids in amylose helices decreased its capacity to bind iodine, and as a result lower absorbance of starch–fatty acid compared to native starch is measured. Therefore, complexing index increases as the iodine capacity decreases. Table 1 presents complexing index and contents of total lipids in tested starch–fatty acid complexes. In both cassava and wheat starches the presence of oleic acid resulted in higher complexing index compared to starch complexes with other fatty acids. Still, complexation of cassava starch with oleic acid was approximately 40% more effective than it was determined in wheat counterpart. The lowest values of CI regardless of starch botanical origin were observed in starch with palmitic acid complexes. Lower susceptibility of palmitic acid (compared to oleic acid) to form complexes with starch was also observed by Annor et al. (2015). These authors observed a significant increase in the CI when unsaturated fatty acids were used for the complexation. Increase in capacity of both starches to complex formation with oleic acid, compared to other fatty acids, may be related to the presence of double bond in its structure. Carbon atoms adjacent to the double bond in oleic acid (cis conformation) may be capable of rotating freely, which may facilitate the complexation of starch with this unsaturated acid in comparison to saturated acids (Karkalas et al. 1995). Among used in the study saturated fatty acids, complexation of both starches with stearic acid resulted in slightly higher values of complexing index in comparison to counterpart complexed with palmitic acid. It may be attributed to stronger hydrophobic interactions between long carbon chains of lipids and the interior of the helix (Arik Kibar et al. 2014).

**Table 1:** Complexing index, total lipids content, and composition of fatty acids in native starches and starch–fatty acid complexes*

Samples	Complexing index (%)	Lipids content (g/100 g d.m. of starch)	Contents of fattyacids (%)		
			Palmitic 16;0	Stearic 18;0	Oleic 18;1n-9
CS	–	0.02 ± 0.01	62.34 ± 0.25	9.95 ± 0.18	10.55 ± 0.47
CS-PA	24.56 ± 1.48	3.29 ± 0.07	96.49 ± 0.06	1.97 ± 0.02^ab^	0.02 ± 0.01^a^
CS-SA	30.13 ± 3.09^a^	4.29 ± 0.85	4.52 ± 0.07^a^	92.34 ± 0.07	0.06 ± 0.01^a^
CS-OA	61.43 ± 3.59	4.21 ± 0.03	4.54 ± 0.01^a^	1.67 ± 0.05^ac^	81.78 ± 0.32
WS	–	0.38 ± 0.07	42.94 ± 0.49	2.05 ± 0.04^ab^	6.90 ± 0.15
WS-PA	29.70 ± 1.21^a^	3.54 ± 0.12	96.38 ± 0.08	2.05 ± 0.10^ab^	0.06 ± 0.01^a^
WS-SA	40.94 ± 0.35	4.09 ± 0.15	4,51 ± 0.09^a^	92.17 ± 0.42	0.12 ± 0.02^a^
WS-OA	42.72 ± 1.29	4.14 ± 0.20	4.52 ± 0.02^a^	1.54 ± 0.01^c^	79.36 ± 0.18

*Mean of three measurements ± standard deviation

Values in the same column with the same letters did not differ significantly (*p* < 0.05)

### Total lipids content and fatty acid composition

The complexation process, apart from increase in CI values, resulted in a significant increase in total lipids contents (Table 1). Complexation of native cassava or wheat starches with oleic and stearic acids increased values of this parameter in a similar range. While, the starch complexes with palmitic acid exhibited lower values of lipids content as compared to the other tested preparations. Out of the native starches the wheat starch was characterized by higher lipids content, which is consistent with other reports (Abera and Rakshit 2003; Sasaki and Matsuki 1998). Differences between lipids contents in tested starch–fatty acids complexes may be attributed to the procedure of complexation. Fatty acids were added to the starch with an equal molar ratio, which in practice resulted in different weights of fatty acids used for complexation. High positive correlations were found between the values of lipids contents in cassava or wheat starch–fatty acid complexes and molar masses of fatty acids used for complexation (*r* = -−0.960 or *r* = -−0.973). Fatty acids used in the study were probably partially complexed with amylose and partially cover the starch surface with a film (Zhou et al. 2007).

The fatty acid compositions of tested starches and starch–fatty acids complexes were compared (supplementary Fig. 1 A–F). In native cassava and wheat starches the dominant fatty acid was palmitic acid (Table 1). In both starches stearic or oleic acids were also determined but in significantly lower quantities, and their proportions were at similar level. After complexation procedure, the percentage share of palmitic, stearic or oleic acids in fatty acid composition of tested complexes increased, which proved that complexation process was effective. The percentage share of palmitic stearic and oleic acids increased approximately up to 96%, 92%, 80%, respectively. In native starches some amounts of polyunsaturated linoleic and linolenic acids were detected. In cassava starch the percentage contribution of those fatty acids were 0.398 ± 0.011% and 1.054 ± 0.025%, respectively. While, in wheat starch linoleic acid constituted 0.027 ± 0.02%, and linolenic 2.334 ± 0.069% of the fatty acids determined in native sample. But still, due to the fact that the native cassava and wheat starches exhibited low content of lipids (0.02% or 0.38%% of d.w., respectively), the amounts of those fatty acids present in sample were very low.

### Water binding capacity and solubility in water

**Fig. 1:**
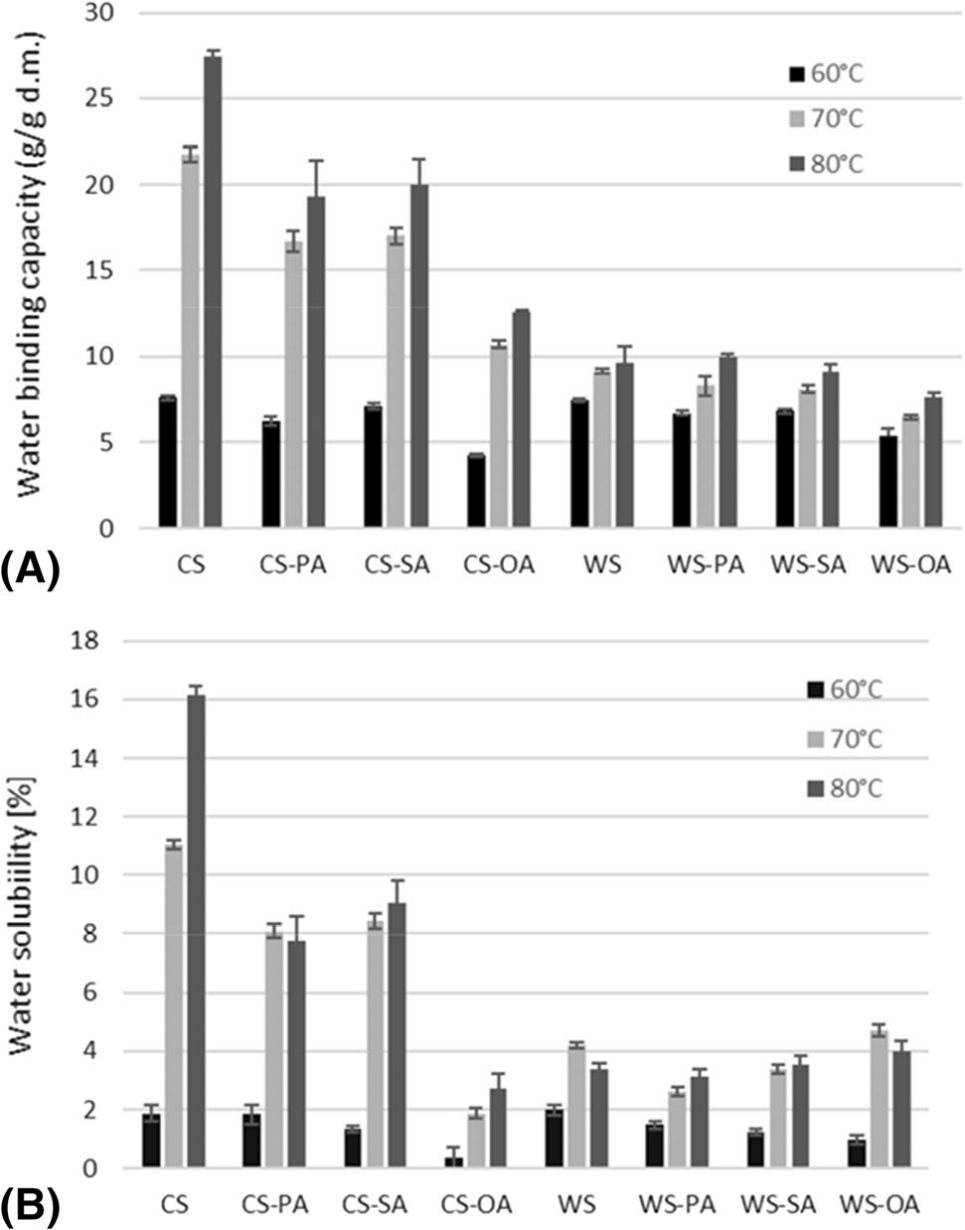
**a** Water binding capacity and **b** water solubility of cassava and wheat starches before and after complexation with fatty acids

The results of measurement of water binding capacity and water solubility of tested starch–fatty acid complexes at temperatures of 60, 70 and 80 °C are collected in Fig. 1. Generally, process of starches complexation with fatty acid resulted in decreased water binding capacity at temperatures of determination, with the greater effect observed in the cassava starch derivatives. Exceptions were observed in water binding capacity values of wheat starch complexes with stearic or palmitic acids at temperature of 80 °C, which did not differ statistically from those determined in native starch. Additionally, in cassava starch and its complexes with fatty acids an increase in temperature of determination resulted in a significant increase in water binding capacity. This might be attributed to their higher amylopectin content (compared to wheat counterparts), which enhances the swelling of starch. These starches are also characterized by lower pasting temperature determined using RVA method, which indicated that they swell and gelatinized at lower temperatures. The lower water binding capacity of wheat starch is probably be due to the lower content of amylopectin in this starch and presence of lipids and mineral elements (in higher amounts than in cassava starch). Obtained results are consistent with data reported by Zi et al. (2019) where water binding capacity values of wheat starches from different cultivars were lower than 10 g/g. In both cassava and wheat starches, complexation with oleic acid resulted in higher decrease of starch water binding capacity, in comparison to other fatty acids used for complexation. It may be related to the higher hydrophobicity of fatty acids with double bonds compared to saturated counterparts (Tang and Copeland 2007). The incorporation of saturated fatty acids, stearic or palmitic, into amylose helices decreased values of this parameter in a similar range. As a result of the increase in temperature of starch suspension amylose is leached from granule interior and cover as a thin layer its surface. In complexed starches those leached amylose helices are partly complexed with fatty acids. The bulky carboxyl groups of fatty acids located outside the helices may also reduce the access of water into starch granule interior thus inhibit starch water binding capacity (Raphaelides and Georgiadis 2006).

Similar to the water binding capacity, the cassava starch complexation with oleic acid decreased significantly its water solubility. It is worth to notice that introduction of palmitic, stearic, or oleic acids into amylose chains reduced the cassava starch water solubility values analysed at 80 °C by approximately 50%, 40% or 80%, respectively. While considering the effect of wheat starch complexation with fatty acids, it has been observed that differences in water solubility between native starch and starch–fatty acid complexes were less noticeable than those observed for cassava starch. Generally, native and complexed with fatty acid wheat starches exhibited relatively low solubility in water in the entire measurement temperature range. Among the tested fatty acids, introduction of palmitic and oleic acids to the native wheat starch significantly reduced its water solubility in all temperatures of determination.

Generally, the water binding capacity of starch is determined, most of all, by amylopectin, whereas its solubility—by amylose (Tester and Morrison 1990). Introduction of fatty acid into amylose helices inhibit the leaching of this starch fraction and therefore delays starch swelling. According to Li et al. (2019) presence of fatty acid in starch structure reduced the osmotic flow of water into starch granule. Furthermore, water solubility of starch is related to the complexing index values (Kaur and Singh 2000). This statement is in good agreement with our results where, both cassava and wheat starch complexes with oleic acid were characterized by larger complexing index values (Table 1) and the lower solubility in water compared to other tested samples.

### Pasting characteristic

The pasting characteristic parameters of native and complexed with fatty acids starches are shown in Table 2. Complexation of cassava starch with fatty acids decreased the peak viscosity of starch approximately by 20%, and also increased the hot paste and final viscosities. The presence of oleic acid in chains of cassava starch amylose resulted in the greatest decrease in PV value and increase in hot paste viscosity, as compared to other fatty acids used in the study. Increase in system viscosity during starch gelatinization (especially at the beginning of the process) is related to leaching of starch components, mainly amylose (Zhou et al. 2007). Therefore, the observed results suggested that starch with the highest complexing index (CS + OA), where the amylose was in the greatest extent bound with the oleic acid showed also significant decrease in PV values. Introduction of stearic or palmitic acids into cassava starch influenced in viscosity changes in a similar range. While considering the effect of wheat starch complexation with fatty on pasting characteristic, it has been observed that only complexation of starch with stearic acid increased peak viscosity of starch pastes. The hot paste viscosities of the tested wheat starch–fatty acids complexes were lower than that of native starch paste, with the greatest effect observed for starch–stearic acid complex. It was different in the case of cassava starch, where starch fatty acid complexes exhibited higher peak viscosities as compared to native starch. This confirms that not only the type of fatty acid used for complexation, but also the botanical origin of starch influences the pasting viscosities. Generally, decrease in PV values observed after starch complexation with fatty acids suggested that the presence of fatty acids in starch structure effectively inhibited its hydratation (Raphaelides and Georgiadis 2006). Additionally, starch granule after complexation with fatty acids may be covered by thin layer of lipids which restrained water transfer into granule. After complexation of the wheat starch with oleic acid the resulting complex was characterized by higher final viscosity than the native starch. On contrary, complexation of this starch with stearic acid resulted in decrease in values of this parameter. These observations prove the effect of fatty acid saturation on values of this parameter. Complexation with saturated stearic acid effectively decreased viscosity values of wheat starch which may be related to inhibitory effect of this fatty acid on WS swelling and hydration (Fig. 1a–b). A diverse effect was observed in case of unsaturated oleic acid addition. Due to the presence of fatty acid, as a guest molecule, the conformation of amylose helices is hydrophobic within the central cavity. In this arrangement hydroxyl groups of amylose are exposed outside the helices and enhance formation of hydrogen bonds which may result in increase in final viscosity of starch–fatty acids pastes (Liang et al. 2002). Generally, the complexation processes of cassava starch caused a significant decrease in breakdown values compared to native starch, which indicated that the rheological stability of starch pastes during their heating and holding at 95 °C increased. While, considering the setback values of cassava starch pastes it was found that complexation with saturated fatty acids increased values of this parameter. While, introduction of unsaturated oleic acid into amylose helices of this starch significantly decreased SB values. This may be due to the fact that part of cassava amylose fraction complexed with oleic acid was unable to associate into crystallites and therefore the complex exhibited lower SB values, which is accordance with complexing index values (cassava starch with oleic acid complex was characterized by the highest complexing index) (Table 1). Generally, the low setback values indicated that starch pastes exhibited lower tendency to retrogradation (Liang et al. 2002). On contrary, complexation of wheat starch with all fatty acid used in the study increased values of breakdown parameter, with the greatest effect observed in the complex of starch with palmitic acid. Zhou et al. (2007) observed that pasting characteristics of the starch significantly changed with the change of amounts of fatty acids complexed with starch.Introduction of oleic or palmitic acids into wheat starch also resulted in an increase in setback values. Generally, the processes of fatty acids complexation significantly increased pasting temperature for wheat starch. Introduction of palmitic or stearic acid caused a similar change in values of this parameter. While, pasting temperature measured for complexes of cassava starch with fatty acids did not differ statistically from non-complexed starch samples. We may presume that decrease in extent of starch granule hydratation, as a result of complexation, indicates an increase in pasting temperature of starch suspension. Therefore, obtained results are in good agreement with determined values of water binding capacity (Fig. 1). High negative correlations were observed between values of P_t_ and WS of cassava and wheat starches (*r* values ranged from −0.942 to −0.788 or from −0.9556 to −0.7822, respectively).

**Table 2:** Pasting properties of native starches and starch–fatty acid complexes*

Samples	PV (mPa s)	HPV (mPa s)	BD (mPa s)	FV (mPa s)	SB (mPa s)	P_t_ (°C)
CS	3650 ± 28	1268 ± 10	2382 ± 18	2086 ± 8	817 ± 18	67.98 ± 0.03^a^
CS-PA	2929 ± 22 ^a^	1489 ± 11^a^	1439 ± 11	2384 ± 28^a^	894 ± 23^a^	68.37 ± 0.32^a^
CS-SA	2930 ± 46^a^	1412 ± 72^a^	1519 ± 27	2278 ± 86^a^	866 ± 13^a^	68.15 ± 0.35^a^
CS-OA	2775 ± 36	1689 ± 84	1086 ± 51	2310 ± 35^a^	620 ± 53	68.35 ± 0.35^a^
WS	1364 ± 25^b^	1003 ± 27	361 ± 9^a^	2127 ± 20^b^	1124 ± 23^b^	83.17 ± 0.53
WS-PA	1374 ± 3^b^	820 ± 17	554 ± 26^b^	2185 ± 33^b^	1365 ± 23	86.42 ± 0.03^b^
WS-SA	1176 ± 7	765 ± 8	411 ± 2A^c^	1916 ± 19	1151 ± 24^b^	87.53 ± 0.03^b^
WS-OA	1430 ± 24^b^	944 ± 18	486 ± 42B^c^	2435 ± 47	1491 ± 32	89.93 ± 0.83

*P_t_,pasting temperature; PV, peak viscosity; HPV, hot paste viscosity; FV, final viscosity; BD, breakdown viscosity; SB, setback viscosity

Mean of three measurements ± standard deviation

Values in the same column with the same letters did not differ significantly (*p* < 0.05)

### Digestibility properties

The starch–fatty acids complexes were analyzed for contents of different starch fractions depending on in vitro digestibility (Table 3). Complexation of cassava starch with stearic acid resulted in a significant decrease in rapidly digestible starch content. While, the introduction of stearic or oleic acids into amylose of cassava starch increased the contents of SDS compared to non-complexed counterpart (two-times or three-times, respectively). The resistant starch contents increased with complexation of native cassava starch with saturated palmitic or stearic acids. Similar effect of palmitic and stearic acids on cassava starch susceptibility to enzymatic hydrolysis was observed by Ai et al. (2013). Interestingly, while considering the contents of nutritionally important fractions in gelatinized wheat starch and its complexes with fatty acid it was found that introduction of tested fatty acids into amylose helices increased content of RDS. The slowly digestible fraction contents of wheat starch–fatty acid complexes were lower than for the native starch. Similar trend was observed by Wang et al. (2016) who demonstrated that wheat starch complexes with fatty acids display higher susceptibility to enzymatic hydrolysis within 0–40 min of digestion. The lowest SDS was shown by complex of starch with oleic acid followed by stearic and palmitic acids complexes. Whereas, RS contents in wheat starch complexes with stearic and oleic were significantly higher than those of native starch or its complex with stearic acid. Among fatty acids used in the study for complexation only palmitic acid did not increase contents of SDS or RS fractions in wheat starch, and therefore did not reduce its susceptibility to enzymatic hydrolysis. The effect of fatty acids complexation on the starch digestibility was investigated also by Kawai et al. (2012). Authors demonstrated that introduction of oleic acid into starch structure decreased the hydrolyzed starch content in obtained starch derivatives, with the major effect on content of starch hydrolyzed within first 20 min of digestion. Additionally, an inhibition effect of oleic and palmitic acids on the enzymatic hydrolysis of pure amylose and starch was observed also by Radhika et al. (2008). Starch enzymatic hydrolysis is a several-stage reaction involves an enzyme diffusion, its adsorption on substrate surface, and ultimately catalysis of glycosidic bonds hydrolysis (Moreira et al. 2012). Obtained results suggested that presence of fatty acids in starch structure may reduce the enzyme access.

**Table 3:** RDS, SDS, and RS values for cassava and wheat starches before and after complexation with fatty acids*

Samples	RDS (%)	SDS (%)	RS (%)
CS	66.90 ± 2.03^a^	3.20 ± 0.71^a^	29.91 ± 2.65
CS-PA	64.78 ± 0.17^a^	2.13 ± 0.68^a^	33.52 ± 0.65^a^
CS-SA	61.35 ± 0.32	6.22 ± 0.19^b^	32.44 ± 0.13^a^
CS-OA	64.70 ± 0.34^a^	10.69 ± 0.31^c^	24.79 ± 0.32
WS	73.63 ± 1.40	16.93 ± 1.17	9.44 ± 0.14^c^
WS-PA	78.06 ± 0.96^b^	11.45 ± 1.52^c^	9.65 ± 0.69^c^
WS-SA	77.89 ± 1.27^b^	6.53 ± 0.80^b^	15.59 ± 0.57^b^
WS-OA	78.91 ± 0.17^b^	3.46 ± 1.84^a^	17.63 ± 1.67^b^

*RDS, rapidly digestible starch; SDS, slowly digestible starch; RS, resistant starch

Mean of three measurements ± standard deviation

Values in the same column with the same letters did not differ significantly (*p *< 0.05)

### Rheological measurements

Pastes formed by cassava starch complexed with fatty acids exhibited lower values of shear stresses in the examined shear rate range compared to the system prepared from native starch (supplementary Fig. 2a–b). Additionally, introduction of oleic acid into cassava starch resulted in the greatest decrease in values of shear stress. When comparing the flow curves of wheat starch systems it is evident that paste formed by complex of starch with stearic acid showed the lowest values of shear stress. But still, systems prepared with native wheat starch and its complexes with stearic or palmitic acids did not differ between each other. Values of Power-Law Model used to describe the experimental flow curves are collected in Table 4. Pastes prepared from tested starch–fatty acid samples pastes were non-Newtonian, pseudoplastic liquids. The high values obtained for the coefficient of determination confirmed the suitability of the used model in fitting the present data. Consistency coefficient (*K*) values corresponds to the apparent viscosity changes of the tested starch pastes. Introduction of fatty acids into native starches in most cases resulted in higher values of consistency coefficient. An exceptions were the pastes of wheat starch complexed with palmitic or stearic acids which did not differ statistically in values of this parameter from uncomplexed starch. It was concluded that pastes formed by starches complexed with oleic acid were more viscous compared to native counterpart. Their *K* values were about two times greater than those determined for native starches. Obtained results are in good agreement with final viscosity values determined using RVA (Table 2). The flow index (*n*) of all tested samples was found to be less than one (Table 4), which depicted that the pastes were pseudoplastic fluids. While, considering the values of flow behaviour index of cassava starch pastes decrease in their pseudolasticity was observed as a result of complexation process. Cassava starch complexes with palmitic and stearic acids were characterized by similar values of this parameter. It is worth to notice that, the presence of oleic acid in both starches used in the study (wheat and cassava) resulted in a significant decrease in the flow index value. Pastes prepared from these starches were more shear thinned than the rest samples and exhibited smoother flow pattern (Zhou et al. 2007). After wheat starch complexation with palmitic and stearic acids, its value did not differ significantly from *n* of the native starch paste. Generally, cassava starch pastes were less pseudoplastic (exhibited higher flow index values) indicating more resistance to shearing forces compared to wheat starch pastes.

**Table 4:** Parameters of the power-law’s model describing pastes prepared from cassava and wheat starches before and after complexation with fatty acids.*

Samples	*K* (Pa s^n^)	*n* (–)	*R*^2^ (–)
CS	13.28 ± 0.42	0.58 ± 0.02	0.986
CS-PA	17.61 ± 0.65^a^	0.51 ± 0.01^a^	0.996
CS-SA	17.95 ± 1.67^a^	0.51 ± 0.02^a^	0.983
CS-OA	33.77 ± 2.23	0.36 ± 0.01	0.983
WS	27.75 ± 0.93^bc^	0.37 ± 0.01^b^	0.993
WS-PA	30.34 ± 7.90^b^	0.35 ± 0.01^b^	0.994
WS-SA	23.20 ± 2.12^c^	0.38 ± 0.01^a^	0.994
WS-OA	50.35 ± 1.80	0.26 ± 0.03	0.997

*K, consistency index; *n,* flow behaviour index; R^2^, determination coefficient

Mean of three measurements ± standard deviation

Values in the same column with the same letters did not differ significantly (*p* < 0.05)

## Conclusion

Performed wheat and cassava starch complexation with palmitic, stearic or oleic acids was effective as was corroborated by determinations of complexing index, lipids content and fatty acid composition. In both cassava and wheat starches the presence of oleic acid resulted in higher complexing index compared to starch complexes with other fatty acids. Procedure of cassava starch complexation with oleic acid decreased in the highest extent its water binding capacity and solubility in water. An increase in final viscosity and rheological stability of cassava starch pastes was observed as a result as a result of cassava starch complexation with all fatty acids used in the study. Pasting temperatures of wheat starch complexes with fatty acids were higher compared to non-complexed counterpart with the greatest effect observed for starch with oleic acid complex. In most cases complexation of starches with fatty acids contribute to an increase in contents of resistant starch fraction. Procedure of oleic acid complexation increased by twice the values of consistency coefficient and significantly decreased flow index of both native wheat and cassava starches.

## Supplementary Information

Below is the link to the electronic supplementary material.

Supplementary file1 (DOCX 135 kb)
