# Supplementary material for: The effect of cassava and wheat starches complexation with selected fatty acids on their functional properties
Source: J Food Sci Technol. 2021 Jun 4;59(4):1440–9. doi: 10.1007/s13197-021-05153-x (PMC8882517; doi:10.1007/s13197-021-05153-x)
Supplement: Supplementary file 1 — Supplementary file1 (DOCX 135 kb) [file 13197_2021_5153_MOESM1_ESM.docx]

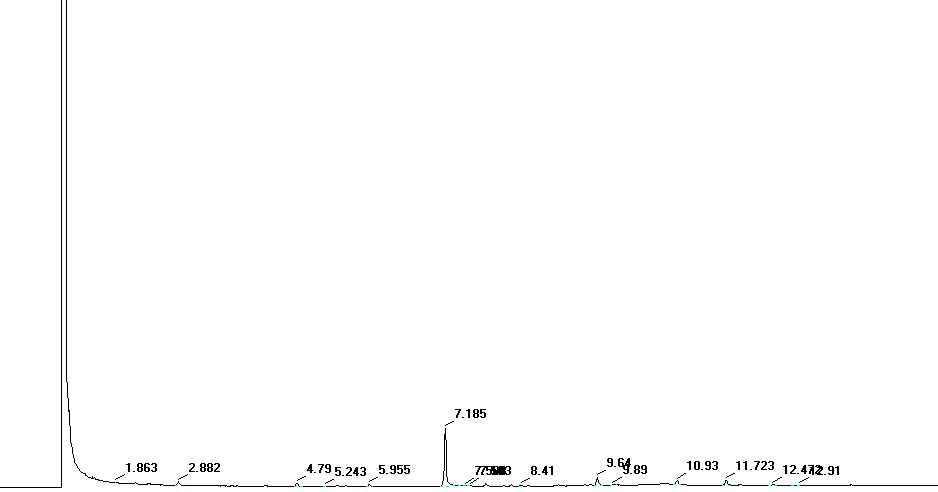


(A)


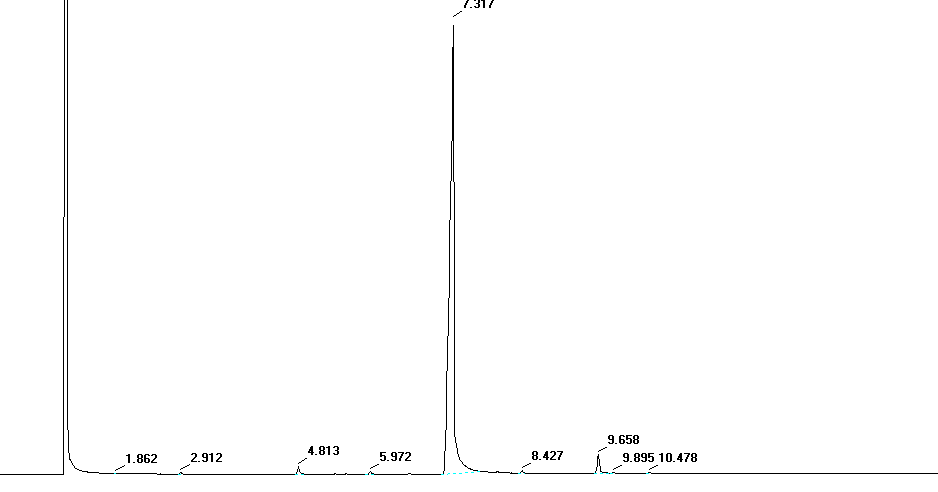


(B)


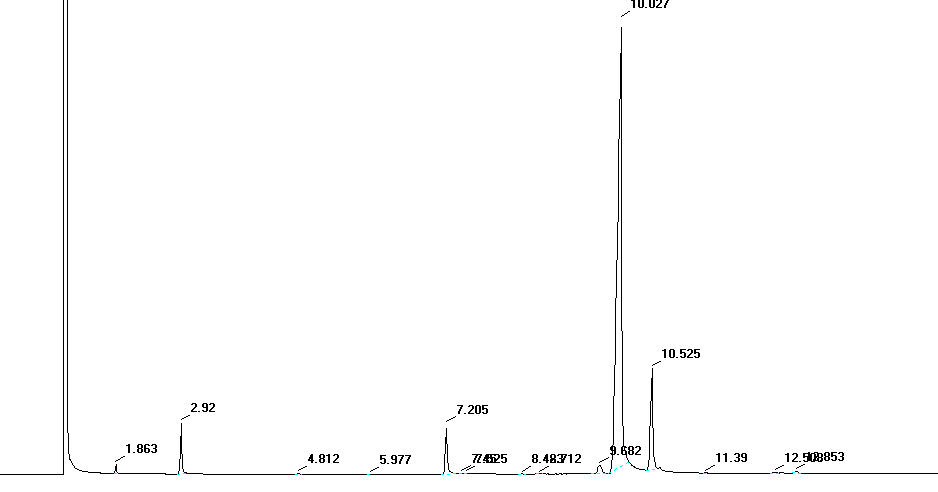


(C)

(A)


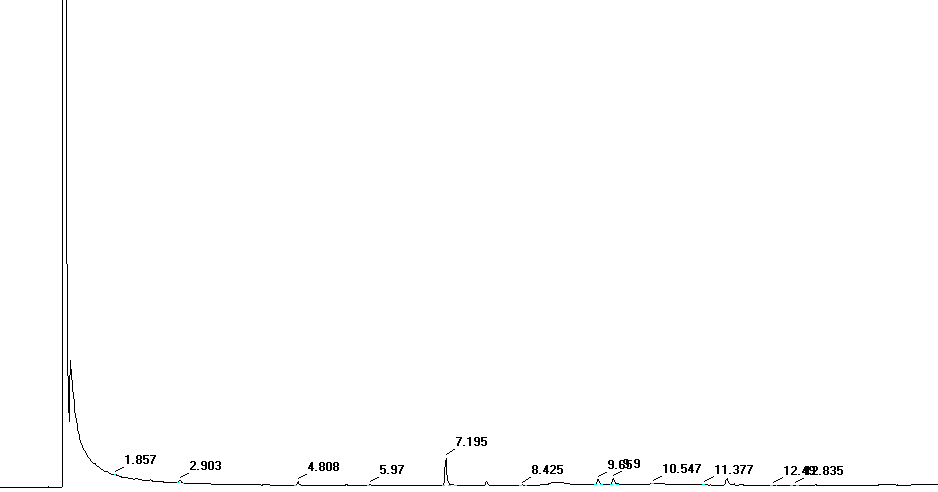


(D)


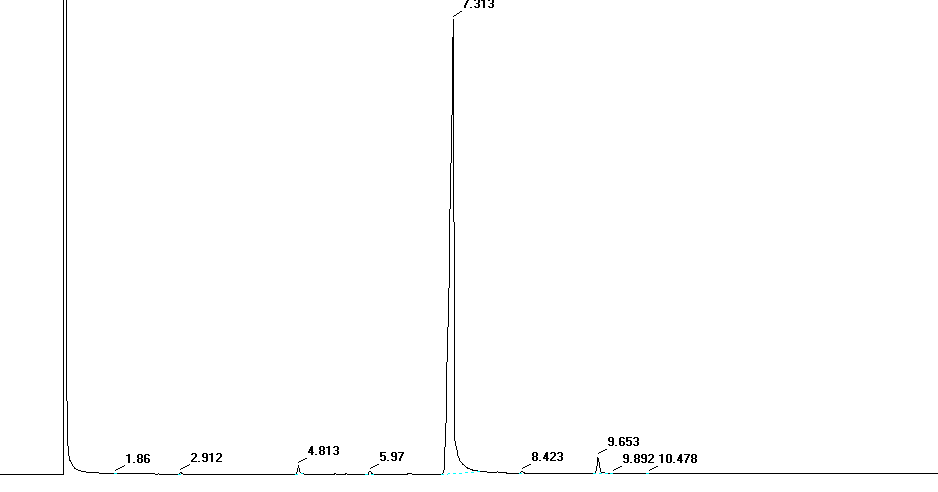


(E)


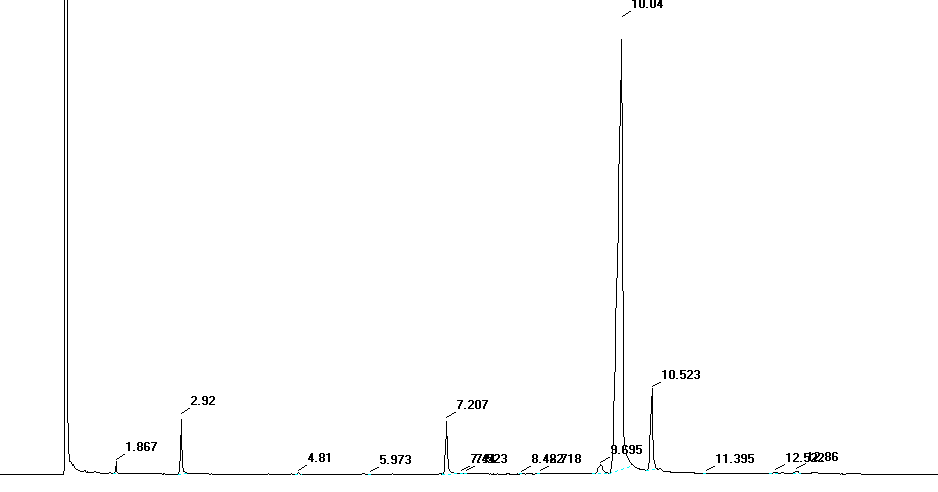


(F)

**Supplementary Figure 1 (A-F).** Gas chromatography profiles of: (A) WS, (B) WS-PA, (C) WS-OA, (D) CS, (E) CS-PA, (F) CS-OA starches.


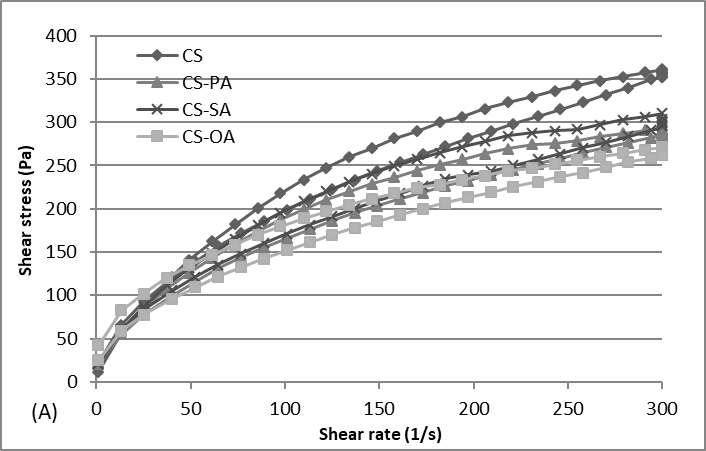


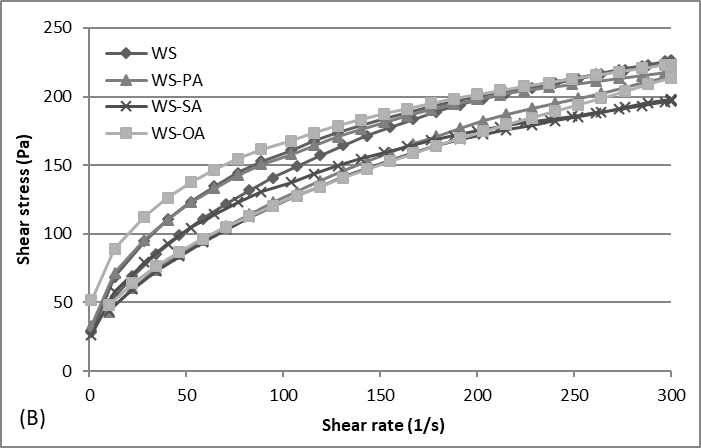


**Supplementary Figure 2 (A-B).** Flow curves of pastes formed by (A) wheat and (C) cassava starches before and after complexation with fatty acids.
